# A Competitor's Point
*Being a letter to the Chairman and members of the East Sussex Hospital, Hastings, of February 16, 1912. It was referred to in Mr. Henman's letter published in our issue of last week, p. 615.


**Published:** 1912-03-23

**Authors:** William Henman


### A COMPETITOR'S POINT.*

Gentlemen,—Trusting that past experience with the existing hospital would compel every precaution being taken to secure a design which would meet modern hospital requirements in a practical and sanitary manner, I ventured to apply experience, gained during the last thirty years in the design and construction of a number of provincial hospitals, to solving the somewhat intricate problem of planning a hospital to detailed requirements on a difficult site.

Realising that others, capable as myself, would be competing, I was prepared to accept the decision, which presumably was acquiesced in by your assessor, until I had the opportuaity to-day of examining the selected plans published in the *Builder*. So faulty are they in so many and important particulars that, in the interest of the undertaking, I respectfully submit it would be advisable, before instructions are given for carrying them into execution, that the following questions should receive most careful consideration :—

1. Did not the conditions properly limit the height of pavilions to two storeys?
2. Are the gradients of roadway, to the casualty and in-patients' entrance, and to the main entrance to administration building, practicable?
3. Is it a satisfactory arrangement to have the casualty department and in-patients' receiving-room so far from the out-patients' department? In provincial hospitals new patients frequently mistake one for the other, and it is not an unusual occurrence for some to wait for hours in the wrong place without receiving attention, when one department is away from the other.
4. Are the casualty and in-patients' receiving-room arranged so that patients on ambulance waggons or stretchers can conveniently be received or transferred to the wards?
5. How could a patient be conveniently conveyed on ambulance trolley or stretcher from the out-patient department to the wards?—such occasionally becomes necessary.
6. Where are the baths, lavatories and w.c.s, sinks, etc., for the two eye-wards, and for the four- and two-bed surgical and medical male and female wards?
7. Where is bath accommodation for gynæcological ward?
8. Where is bath accommodation for two isolation wards?
9. How can patients on ambulance trolleys or stretchers be conveyed into the two- and four-bed wards?
10. Is it advisable that staircases should give air communication between the two and three storeys of main pavilions?
11. Where are the cleaners and trolley rooms to any of the five floors of wards?
12. What is the practical utility in providing external stairways for escape in case of emergency if it is impossible to convey patients in beds or on stretchers to and down same?
13. How is the out-patients' department to be economically supplied with hot water and heating with boilers at a considerably higher level?

* Being a letter to the Chairman and members of the East Sussex Hospital, Hastings, of February 16, 1912. It was referred to in Mr. Henman's letter published in our issue of last week, p. 615.

14. How can foul linen from the wards, etc., be conveniently conveyed to the disinfector and thence to the laundry, or even to the laundry direct from the wards, except past kitchen and store-rooms?

15. Is it convenient, when medicines are required for the wards from the dispensary, that they should have to be conveyed up a stairway and a considerable distance along a corridor to the lift, and then, for most of the wards, again along a corridor?

16. Is it a sanitary arrangement that foul water from the operating department should have to be discharged into the small areas, three storeys deep, near the kitchen department, and thence to be conveyed *under* the buildings?

17. How can foul water and rain-water, as well as snow occasionally, otherwise be removed from these small areas the depth of three storeys?

18. Is it desirable that house coal should be shot down in a narrow passage close to kitchen entrance off an enclosed yard, too small in which to turn a cart round?

19. Is the provision for getting in coal for boilers practicable?

20. Is it good to shut out sunlight by wide balconies at ends of wards, and to have the sides of wards overshadowed by projecting sanitary turrets?

21. Is it advisable that tradesmen and stores should enter close to the mortuary and out-patients' department?

22. Is the isolation block conveniently placed for supplying meals? How would patients be conveyed there, and how would they be visited by medical men, particularly in the event of the additional pavilions being erected?

23. Will the ophthalmic surgeon consider it a convenient arrangement that he will have to traverse 250 feet of corridor to get from three male to three female patients?

24. How is a corpse to be conveniently transferred from a ward to the mortuary?

25. Is the requirement reasonably fulfilled that the hospital should be designed so that portions can be erected as funds permit?

The foregoing questions imply serious faults in planning which appear in the selected design, not one of which, I can confidently state, is to be found in the design I had the honour of submitting, and I doubt if many other designs so flagrantly disregard undoubted essentials in hospital planning.

Whatever may be the result of investigation on the above points, I trust it may, at least, prevent heavy expenditure on faulty lines.—I am, gentlemen, your obedient servant, (Signed) William Henman, F.R.I.B.A.

